# Supplementary material for: Rashba splitting in organic–inorganic lead–halide perovskites revealed through two-photon absorption spectroscopy
Source: Nat Commun. 2022 Jan 25;13:483. doi: 10.1038/s41467-022-28127-9 (PMC8789784; doi:10.1038/s41467-022-28127-9)
Supplement: Supplementary file 1 — Supplementary Information [file 41467_2022_28127_MOESM1_ESM.pdf]

Rashba Splitting in Organic-Inorganic Lead-Halide Perovskites Revealed through Two-Photon Absorption Spectroscopy

Supplementary Note 1. Symmetry Analysis of One- and Two-Photon Accessible Exciton States

Here we present in detail the group symmetry analysis of available pathways for one- and two-photon transitions for example crystal systems relevant to the observation of the presence or absence of TP-PLE in the exciton range of material systems described in the main text. Two-photon accessible states require that an intermediate state be accessible by a one-photon transition from the crystal ground state and further that the final state be accessible by a one-photon transition from the intermediate states. These processes are described by the first and second transition matrix elements between such states. Whether a certain transition is allowed is determined by consideration of the point symmetry of the system and the irreducible representations (irreps) of the conduction and valence band states involved. The irreducible representations in the discussion to follow are labeled according to the KDWS scheme[1]. The strong spin-orbit interaction is accounted for by using the double group irreps to describe the carrier states prior to constructing the exciton states. The irrep of the exciton state, Γ_{exc}, can be constructed as $\Gamma_{\mathrm{exc}} = \Gamma_{\mathrm{c}} \otimes \Gamma_{\mathrm{h}} \otimes \Gamma_{\mathrm{env}}$, where Γ_{c} and Γ_{h} are the irreps of the electron and hole Bloch functions and Γ_{env} is the irrep of the exciton envelope function. Likewise, the symmetry properties of the transition matrix element can be determined by assigning the component of the dipole operator, (which, as a polar vector, transform like the coordinates x,y,z), to the irreps of the corresponding point group. For a given exciton state to be accessible by one- or two-photon absorption, its irrep must be present in the decomposition of the appropriate transition matrix element. These are proportional to the dipole components x,y,z for one photon absorption, or products of these components for the two photon processes.

In Supplementary Figure 1 we present the transition sequences for a cubic system with O_h point symmetry group, which possesses inversion symmetry. Here, parity is a good quantum number. Thus, the ground state and two-photon accessible states need to have even total parity, whereas the one-photon accessible states need to have odd total parity (odd parity Bloch function with even parity envelopes). The result is that states excited by two-photon excitation cannot relax to the ground state by emission of a single photon, leading to the separation between the TP-PLE spectra and the one-photon absorption spectra onsets observed in Figures 2a and 2c in the main

text. The same strict separation of exciton envelopes by parity is observed in the inversion symmetric monoclinic phase with C_{2h} point symmetry (Supplementary Figure 2).

In systems that lack inversion symmetry this situation changes through the relaxation of the parity selection rule. In Supplementary Figure 3 we show the available transition pathways for a cubic system subject to symmetry breaking along the crystallographic z-axis, resulting in the lowering of the symmetry to C_{4v}. For simplicity we limit the excitation polarization to the x-y plane. While certain excitons can still be strictly separated into either one-photon or two-photon accessible, we see that two sequential excitations can lead to the population of the Γ_1 state, which may then relax to the ground state by emission of a single photon polarized in the z-direction. The situation is much more dramatic in the inversion asymmetric monoclinic phase with C_s symmetry as shown in Supplementary Figure 4, where all two-photon accessible states are one-photon coupled to the ground state. In these latter two cases, an overlap between the TP-PLE and absorption spectra in the exciton range is expected, consistent with what is observed in Figures 4a,d and Figure 5 in the main text.

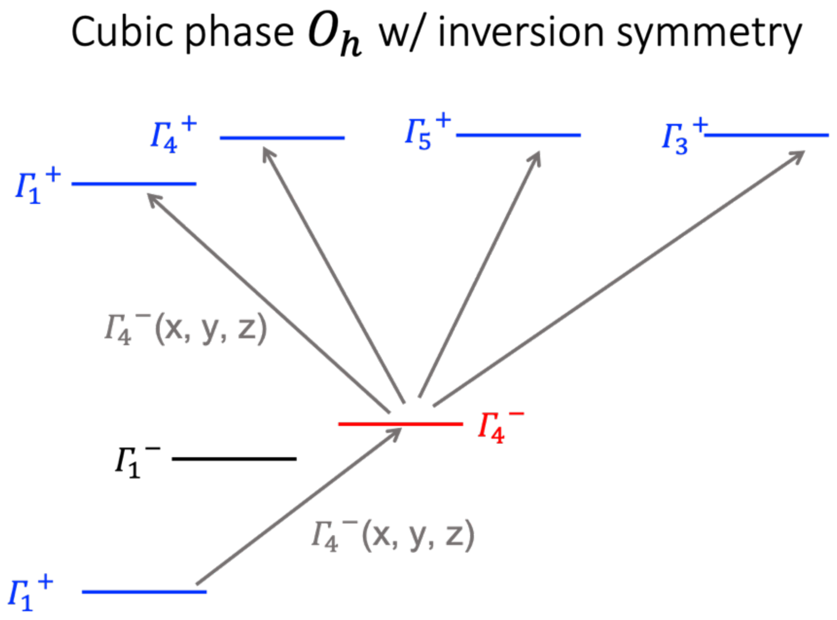

Supplementary Figure 1. | Exciton energy levels and their symmetries for a cubic crystal with inversion symmetric O_h point group. Exciton energy levels of even (blue) and odd (red) total parity states labeled by their irreducible representations using the KDWS nomenclature. The Γ_1^- shown in black is spin forbidden and thus not accessible by one or two-photon transitions. The grey arrows represent single photon dipole transitions and are labeled by the irreducible representation for polarizations along x, y, and z crystal axis.

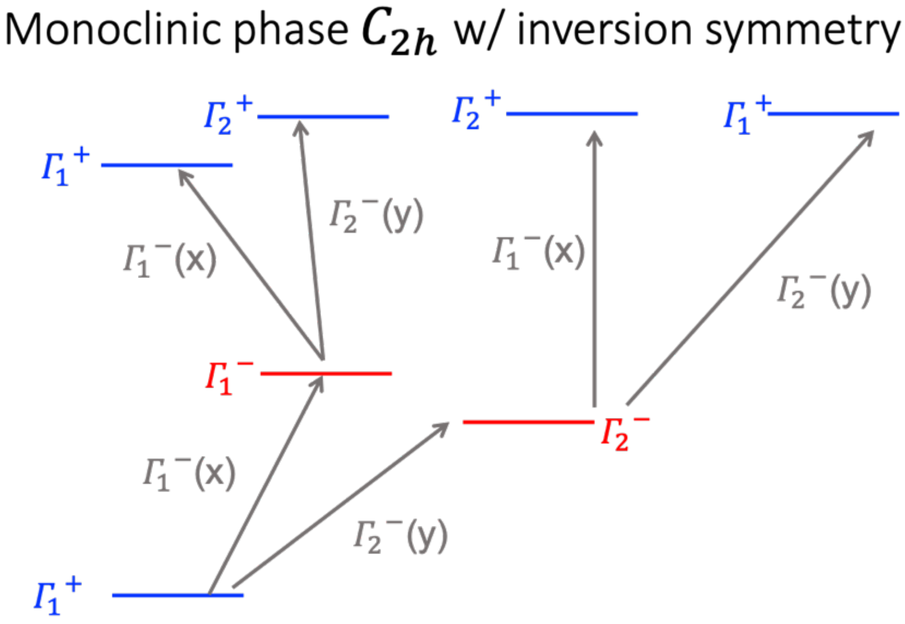

Supplementary Figure 2. l Exciton energy levels and their symmetries for a monoclinic crystal with inversion symmetric C_{2h} point group, with the C_2 rotation axis taken to be the x direction. Exciton energy levels of even (blue) and odd (red) total parity states labeled by their irreducible representations using the KDWS nomenclature. The grey arrows represent single photon dipole transitions and are labeled by the irreducible representation for polarizations of light along x and y crystal axis.

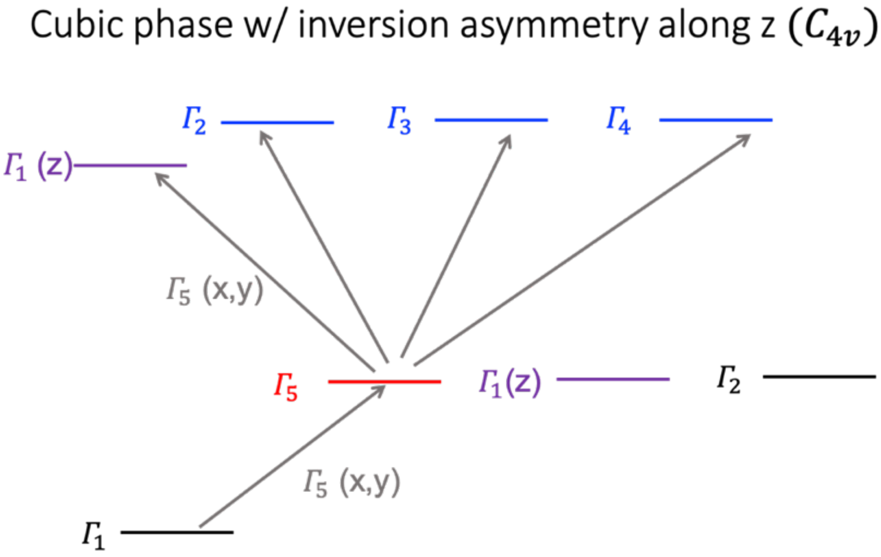

Supplementary Figure 3. l Exciton energy levels and their symmetries for a cubic crystal with broken inversion symmetry along the z-axis (C_{4v} point group). Exciton energy levels of exclusively one-photon allowed (red), exclusively two-photon allowed (blue), and one- and two-photon accessible (purple) states labeled by their irreducible representations using the KDWS nomenclature. The latter are also labeled by the polarization of light by which they are one-photon coupled to the ground state. The Γ_2

shown in black is spin forbidden and thus not accessible by one or two-photon transitions. The grey arrows represent single photon dipole transitions and are labeled by the irreducible representation for polarizations of light along x, y, and z crystal axis.

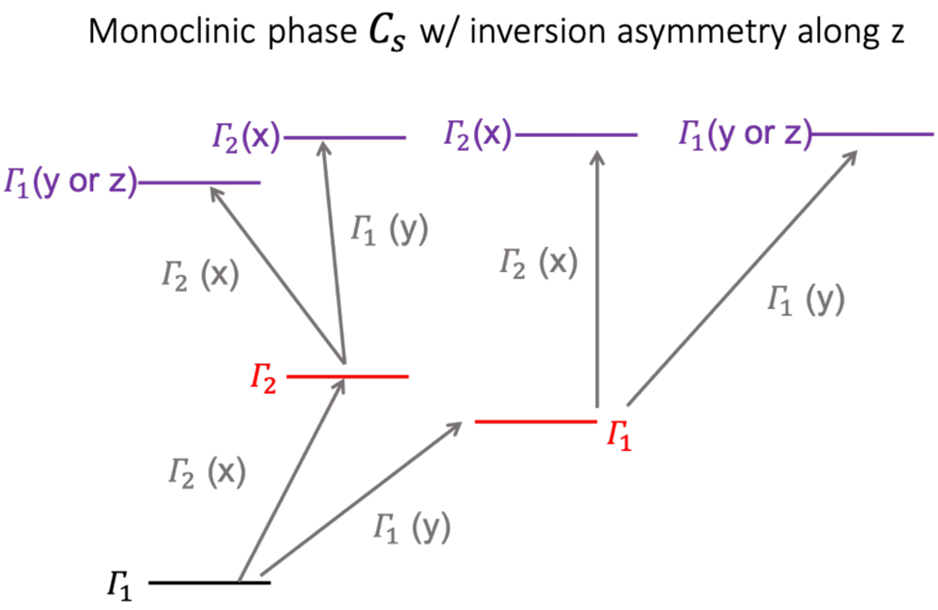

Supplementary Figure 4. | Exciton energy levels and their symmetries for a monoclinic crystal with broken inversion symmetry along the z-axis (C_S point group). Exciton energy levels of exclusively one-photon allowed (red) and one- and two-photon accessible (purple) states labeled by their irreducible representations using the KDWS nomenclature. The latter are also labeled by the polarization of light by which they are one-photon coupled to the ground state. The grey arrows represent single photon dipole transitions and are labeled by the irreducible representation for polarizations of light along x, y, and z crystal axis. The crystal structure is assumed to be the same as in Fig S2 but with the addition of inversion asymmetry along the z-axis.

Supplementary Note 2. Two Photon Absorption of Semiconductors with Rashba Band-Dispersion

A single cartesian component of third-order nonlinear susceptibility that gives rise to two-photon absorption in response to a beam of frequency ω polarized in the same direction as the response can be described[2] by:

$$\chi_{iiii}{}^{(3)}(-\omega;\ \omega,\omega,-\omega) = \frac{N}{\hbar^3}\sum\nolimits_{vnml} \rho_{ll}{}^{(0)}$$
$$\left\{\frac{\mu_{lv}\mu_{vn}\mu_{nm}\mu_{ml}}{[\omega_{vl}-\omega][\omega_{nl}-2\omega][\omega_{ml}-\omega]} + \frac{\mu_{lv}\mu_{vn}\mu_{nm}\mu_{ml}}{[\omega_{nv}-\omega][\omega_{mv}-2\omega][\omega_{vl}+\omega]} \right.$$
$$+ \frac{\mu_{lv}\mu_{vn}\mu_{nm}\mu_{ml}}{[\omega_{nv}-\omega][\omega_{vm}+2\omega][\omega_{ml}-\omega]} + \frac{\mu_{lv}\mu_{vn}\mu_{nm}\mu_{ml}}{[\omega_{nv}-\omega][\omega_{nl}+2\omega][\omega_{vl}+\omega]}$$
$$+ \frac{\mu_{lv}\mu_{vn}\mu_{nm}\mu_{ml}}{[\omega_{vn}+\omega][\omega_{nl}-2\omega][\omega_{ml}-\omega]} + \frac{\mu_{lv}\mu_{vn}\mu_{nm}\mu_{ml}}{[\omega_{nm}+\omega][\omega_{mv}-2\omega][\omega_{vl}+\omega]} \tag{S1}$$
$$+ \frac{\mu_{lv}\mu_{vn}\mu_{nm}\mu_{ml}}{[\omega_{nm}+\omega][\omega_{vm}+2\omega][\omega_{ml}-\omega]}$$
$$\left. + \frac{\mu_{lv}\mu_{vn}\mu_{nm}\mu_{ml}}{[\omega_{ml}+\omega][\omega_{nl}+2\omega][\omega_{vl}+\omega]}\right\}$$

Here N is the total number of electrons, $\rho_{ll}{}^{(0)}$ is the population density of the lth quantum state, μ_{pq}^{i} is the transition moment between the states p and q, and the effects of damping have been neglected. At a given **k**, for a single pair of degenerate conduction and valence bands, the cumulative transition moment pathways for terms that contain a resonance at 2ω are shown in Supplementary Figure 5. Each transition involves terms represented by vertical arrows pointing up or down corresponding to the transition moments μ_{21} and μ_{12}, respectively, as well as circular arrows in the valence or conduction band which correspond to the terms μ_{11} and μ_{22}, respectively. The latter indicate *intra*-band transitions that would be strictly zero for states with definite parity, such as occurs at $\mathbf{k} = 0$ or at other high symmetry points in the Brillouin Zone (BZ). Away from these points they have finite magnitude, and TPA becomes allowed.

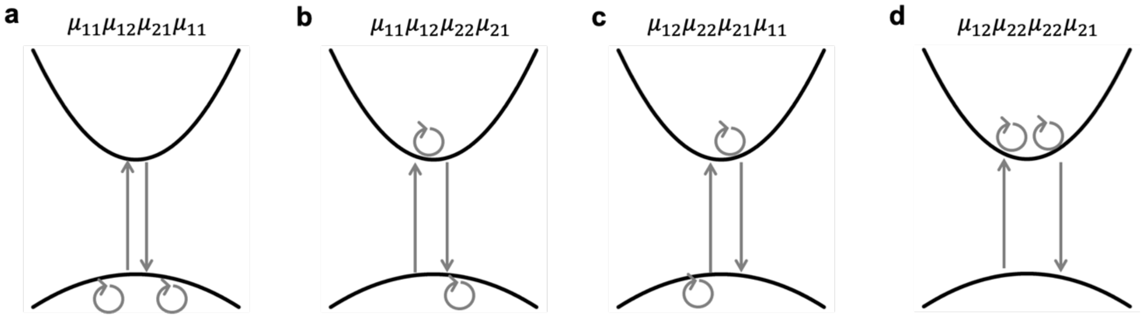

Supplementary Figure 5. | Multi-photon pathway contributions to two-photon absorption for a two-band system. a-d Schematic illustration of the transition moment contributions to two-photon absorption for a two-band system. The grey arrows represent the four transition moment contributions involved in each term. The transition moments for each term are labeled in the top of the figure.

When the Rashba effect breaks the spin degeneracy of the bands, the number of participating states increases from two to four. The result is a large number of additional multiphoton pathways become available to contribute to TPA as shown in Supplementary Figure 6. Labelling the bands in terms of increasing energy as bands 1 (magenta), 2 (green), 3 (blue) and 4 (red), we first note that terms of the kind shown in Supplementary Figure 5 will exist between band 1 and bands 3 $(1 \rightarrow 3)$ and 4 $(1 \rightarrow 4)$ and similarly for band 2 such terms will contribute to transitions $2 \rightarrow 3$ and $2 \rightarrow 4$. In addition to these there will be terms such as those displayed in Supplementary Figure 6 a-j for the transitions $2 \rightarrow 3$ (Supplementary Figure 6 a–e) and $2 \rightarrow 4$ (Supplementary Figure 6 f–j) that involve transitions between bands 3 and 4 as intermediate states and another similar 10 terms in which the initial state rests in band 1 instead (not shown). Furthermore, there will contributions to the transition $2 \rightarrow 3$ that involve transitions between bands 1 and 2 as intermediates such as those shown in Supplementary Figure 6 k-o, as well as similar terms where the final state involved resides in band 4 instead of band 3 (not shown). Lastly, there will be 8 more contributions that involve all four distinct bands in the summation such as those pictured in Supplementary Figure 6 p–w. The strength of each contribution depends on the specific magnitude of each of the transition moments of the form μ_{ij}, which requires accurate knowledge of the form of the wavefunctions associated with each state, but this analysis provides a qualitative picture of how the Rashba splitting results in both TPA at higher energy than in a typical degenerate semiconductor (due to higher energy final states) as well as an increase in the strength of the TPA (due an increase in the number of intermediate states available). In the next section we discuss the quantitative calculation of TPA in the presence of the Rashba effect using $\boldsymbol{k} \cdot \boldsymbol{p}$ theory.

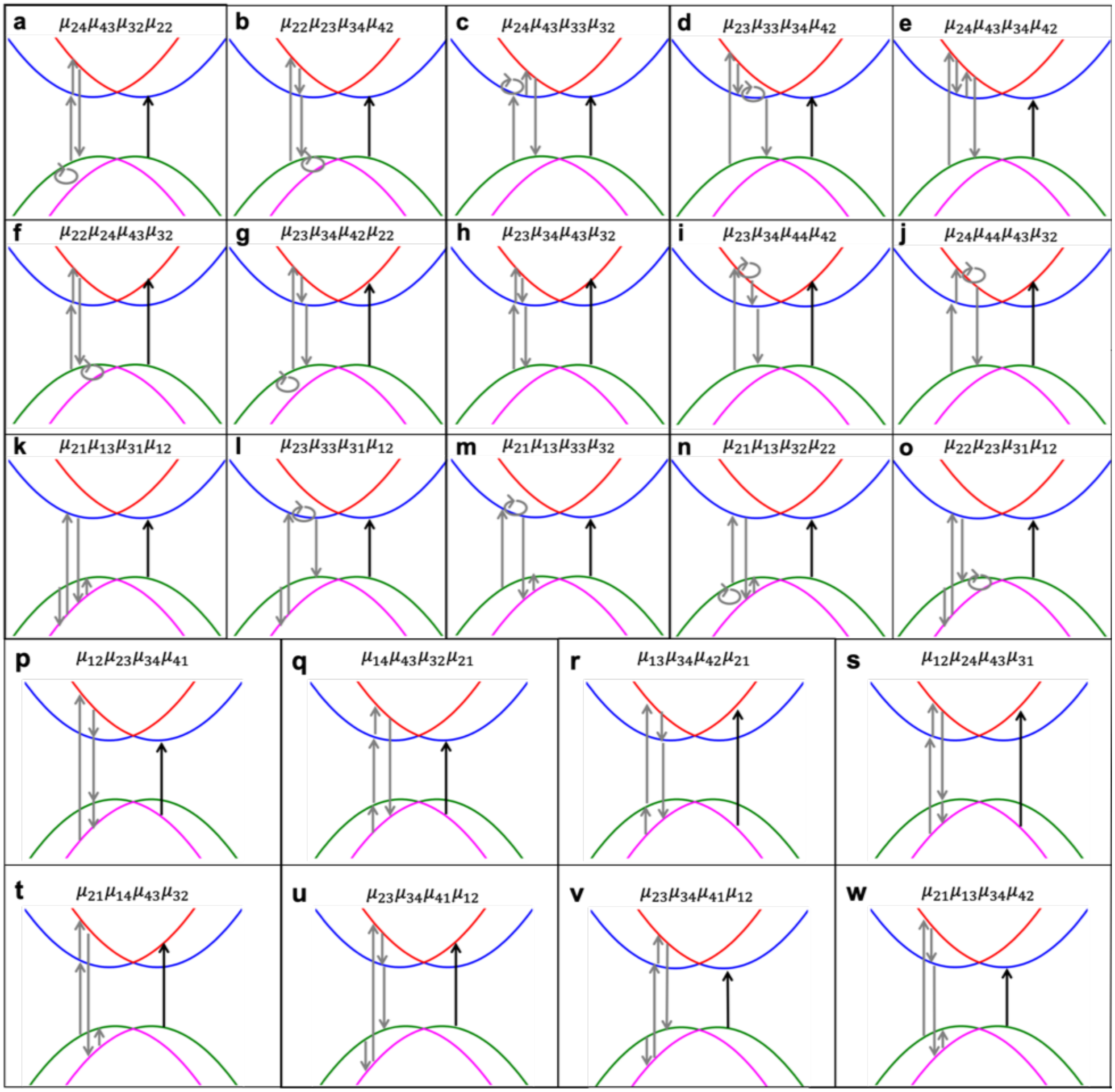

Supplementary Figure 6. | Multi-photon pathway contributions to two-photon absorption for a four-band system. a-e Transitions from band 2 (green) to band 3 (blue) involving bands 2, 3, and 4 (red) as intermediate states. **f-j** Transitions from level 2 to 4 involving bands 2, 3, and 4 as intermediate states. **k-o** Transitions from level 2 to 3 involving bands 1 (magenta), 2, and 3 as intermediate states. **p-w** Transitions involving all four bands as intermediate states. The grey arrows represent the four transition moment contributions involved in each term. The black arrow connects the initial band to the final band. The transition moments for each term are labeled in the top of the figure.

Supplementary Note 3. Calculation of Two-Photon Absorption Spectra

For calculating the two-photon absorption (TPA), we used the second-order perturbation theory. The initial state is the ground state with energy E_i plus two photons and the final state is a real, excited state of the material with energy E_f. Conservation of energy requires $E_f = E_i + 2\hbar\omega$, with $\hbar\omega$ the photon energy. Meanwhile, because the photon momentum is negligible on the scale of the Brillouin Zone (BZ), both initial, intermediate and final states have the same momentum $(k_i = k_t = k_f)$. The intermediate state is a virtual state with energy E_t. The probability of absorbing two photons is

$$W = \frac{2\pi}{\hbar}\left(\frac{4\pi^2 e^4 I_p^2}{n_p^2 c^2 m^4 \omega^4}\right)\int \sum\nolimits_f \left|\sum\nolimits_t \frac{M_{ft}M_{ti}}{E_f - E_t - \hbar\omega}\right|^2 \delta\left(E_f - E_i - 2\hbar\omega\right)\frac{d^3k}{(2\pi)^3} \qquad \text{(S2)}$$

where I_p is the pump intensity, n_p is the refractive index at the pump photon energy, m is the free electron mass, and M_{ft} and M_{ti} are the transition matrix elements

$$M_{ft} = \langle f|\boldsymbol{\varepsilon}\cdot\boldsymbol{p}|t\rangle, M_{ti} = \langle t|\boldsymbol{\varepsilon}\cdot\boldsymbol{p}|i\rangle, \qquad \text{(S3)}$$

with $\boldsymbol{\varepsilon}$ the polarization vector of the pump beam, and **p** the momentum operator. The TPA coefficient $\beta(\omega)$ is defined as

$$\beta(\omega) = \frac{2\hbar\omega W}{I_p^2} \qquad \text{(S4)}$$

in units of cm/MW. The band structure of the perovskites has been obtained based on the $\boldsymbol{k}\cdot\boldsymbol{p}$ model[3].

In Supplementary Figure 7 we demonstrate the results of this calculation for $MAPbBr_3$. The calculations do not include any exciton effect (see Supplementary Figure 11). We see that β increases from zero and reaches a maximum at around $2\hbar\omega/E_G = 1.4$ and then decreases with increasing photon energy.

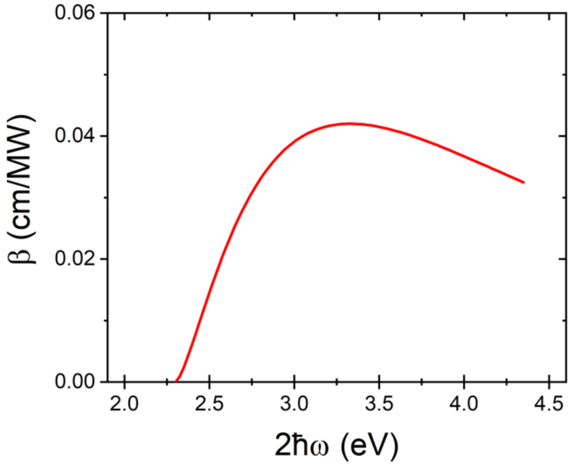

Supplementary Figure 7. | TPA spectrum of MAPbBr3 with final states limited to the first conduction band.

In the above calculations we have neglected the Rashba effect. Incorporating these effects, the Hamiltonians for the conduction and valence bands can be written,

$$H_{c\boldsymbol{k}} = \frac{\hbar^2}{2m_e}\left(k_x^2 + k_y^2 + k_z^2\right) + \alpha_c\left(k_y\sigma_x - k_x\sigma_y\right) \tag{S5a}$$

$$H_{v\boldsymbol{k}} = -E_G - \frac{\hbar^2}{2m_h}\left(k_x^2 + k_y^2 + k_z^2\right) + \alpha_v\left(k_y\sigma_x - k_x\sigma_y\right) \tag{S5b}$$

The Rashba effect lifts the spin degeneracy and splits the lowest conduction band (c) into upper and lower bands ($c\pm$) with energy dispersion

$$E_{c\boldsymbol{k}}^{\pm} = \frac{\hbar^2}{2m_e}\left[(k_\perp \pm k_{c0})^2 + k_z^2\right] - \theta_c^0 \tag{S6a}$$

where $k_{c0} = |\alpha_c| m_e/\hbar^2$ and $\theta_c^0 = \hbar^2 k_{c0}^2/2m_e$. Similarly, the valence band (v) is split into upper and lower bands ($v\pm$) with dispersion,

$$E_{v\boldsymbol{k}}^{\pm} = -E_G + \theta_v^0 - \frac{\hbar^2}{2m_h}\left[(k_\perp \pm k_{v0})^2 + k_z^2\right] \tag{S6b}$$

where $k_{v0} = |\alpha_v| m_h/\hbar^2$ and $\theta_c^0 = \hbar^2 k_{v0}^2/2m_h$. For TPA, the initial state is still the completely occupied valence bands plus two photons to be absorbed, but now the final state may take on four possibilities with an electron in either ($c\pm$) and a hole in either ($v\pm$). The intermediate

state will also have the four possibilities, plus one photon to be absorbed. In addition, the matrix elements must be carefully evaluated.

For the electric-dipole transition with the electron-photon interaction expressed as

$$P_v = -\frac{e}{mc}\boldsymbol{A}\cdot\boldsymbol{p} = -\frac{e}{c}\boldsymbol{A}\cdot\boldsymbol{v} \tag{S7}$$

In the presence of the Rashba effect, the velocity operator for the conduction and valence bands must be reevaluated from the Hamiltonians $H_{c\boldsymbol{k}}$ and $H_{v\boldsymbol{k}}$.

$$\boldsymbol{v}_c = \dot{\boldsymbol{r}} = \frac{\mathbf{1}}{i\hbar}[\boldsymbol{r}, H_c] = \frac{\hbar}{2\sqrt{m_e}}(e_+k_- + e_-k_+) + i\sqrt{2}\frac{\hbar k_{0c}}{m_e}(e_-\sigma_+ - e_+\sigma_-) \tag{S8a}$$

$$\boldsymbol{v}_v = \dot{\boldsymbol{r}} = \frac{\mathbf{1}}{i\hbar}[\boldsymbol{r}, H_v] = \frac{\hbar}{2\sqrt{m_h}}(e_+k_- + e_-k_+) + i\sqrt{2}\frac{\hbar k_{0v}}{m_h}(e_-\sigma_+ - e_+\sigma_-) \tag{S8b}$$

where $e_\pm = \frac{1}{\sqrt{2}}(e_x \pm ie_y)$ and $k_\pm = k_x \pm ik_y$ and $\sigma_\pm = \sigma_x \pm i\sigma_y$. The first two terms are the common velocity, $\boldsymbol{v}_{c(v)} = \hbar\boldsymbol{k}/m_{e(h)}$ in a parabolic band, which preserves (pseudo)spin. The second terms, however are induced by the Rashba effect.

Labeling the bands in order of increasing energy as 1-4, i.e. $v_- = 1$, $v_+ = 2$, $c_- = 3$, and $c_+ = 4$, (Supplementary Figure 8) and denoting the four final states by the location of the resulting electron and hole, (e.g. (3, 2) for the final state with electron in band 3, and hole in band 2), we have the four possibilities (3, 1), (3, 2), (4, 1) and (4, 2). The transition rate for a given state, say (4,1) is then

$$W_{(4,1)} \propto \left|\sum_{t=1}^{4}\frac{\langle 4|\boldsymbol{\varepsilon}\cdot\boldsymbol{p}|t\rangle\langle t|\boldsymbol{\varepsilon}\cdot\boldsymbol{p}|1\rangle}{E_t - E_1 - \hbar\omega}\right|^2 \delta(E_4 - E_1 - 2\hbar\omega) \tag{S9}$$

In each of the four terms, one of the two matrix elements represent an *inter*-band while the other represents an *intra*-band transition. The inter-band terms, denoted as p_{cv}, are large and independent of the Rashba effect. Whereas the *intra*-band terms depend on the Rashba effect which can be evaluated using the velocity operators. Note that these velocity operators include an additional "spin-flip" channel. In the absence of the Rashba effect the matrix elements $\langle 4|P_v|3\rangle$ and $\langle 2|P_v|1\rangle$ are null due to the opposite spins in the conduction and valence sub-bands. In the presence of the Rashba effect that mixes spin and orbital momentum, these additional "spin-flip" terms become available.

After evaluating the individual matrix elements, we can express the transition rate $W_{(4,1)}$ as

$$W_{(4,1)} \propto |p_{cv}|^2 \left[\left(\frac{k_\perp}{\omega m_{cv}} \right)^2 + \left(\frac{k_{0c}}{m_e(\omega - E_{43}/\hbar)} - \frac{k_{0v}}{m_h(\omega - E_{21}/\hbar)} \right)^2 \right] \quad \text{(S10a)}$$

where $k_\perp = \sqrt{k_x^2 + k_y^2}$ and $E_{ij} = E_i - E_j$. Similarly, for the other three transitions,

$$W_{(3,1)} \propto |p_{cv}|^2 \left[\left(\frac{k_\perp}{\omega m_{cv}} \right)^2 + \left(\frac{k_{0c}}{m_e(\omega + E_{43}/\hbar)} + \frac{k_{0v}}{m_h(\omega - E_{21}/\hbar)} \right)^2 \right] \quad \text{(S10b)}$$

$$W_{(4,2)} \propto |p_{cv}|^2 \left[\left(\frac{k_\perp}{\omega m_{cv}} \right)^2 + \left(\frac{k_{0c}}{m_e(\omega - E_{43}/\hbar)} + \frac{k_{0v}}{m_h(\omega + E_{21}/\hbar)} \right)^2 \right] \quad \text{(S10c)}$$

$$W_{(3,2)} \propto |p_{cv}|^2 \left[\left(\frac{k_\perp}{\omega m_{cv}} \right)^2 + \left(\frac{k_{0c}}{m_e(\omega + E_{43}/\hbar)} - \frac{k_{0v}}{m_h(\omega + E_{21}/\hbar)} \right)^2 \right] \quad \text{(S10d)}$$

Since the photon energy is much higher than the Rashba splitting we see that in $W_{(4,1)}$ and $W_{(3,2)}$ the extra contributions from the spin-flip channels cancel out, i.e. destructive interference. On the other hand, the contributions from the extra channels reinforce each other for $W_{(3,1)}$ and $W_{(4,2)}$. Consequently, $W_{(3,1)}$ and $W_{(4,2)}$ are much stronger. This can loosely be interpreted as transitions for $W_{(3,1)}$ and $W_{(4,2)}$ having the correct spin alignments whereas for $W_{(4,1)}$ and $W_{(3,2)}$ have the opposite spin alignments.

The result is that new final states are available at higher energy above E_G. In accordance with the effective mass model[3] it can be shown that in the particular case of hybrid perovskites ${\alpha_R}^c \approx 2{\alpha_R}^v$. Evaluating eqs. S10(a – d) we find two distinct manifestations of the contribution of the Rashba effect to the TPA spectrum. When the effective masses of the VB and CB are approximately equal, $W_{(4,1)}$ and $W_{(3,2)}$ are relatively weak while the contributions from $W_{(3,1)}$ and $W_{(4,2)}$ are much stronger and dominate the TPA spectrum. The onset energies for these transitions occur at $\boldsymbol{k} = 0$, at the Dirac point. Because the terms $|p_{cv}|^2$ are proportion to $\boldsymbol{k}^2$, the TPA bands grow slowly and the transitions are spread out in k-space and energy resulting in a broad TPA feature.

The value of the Rashba parameter may thus be obtained from direct comparison to the model calculations as was done in Figure 4a of the main text. The effective masses are to $m_e = m_h = 0.10m$. The fitting results in values for the Rashba splitting energy of ${E_R}^{c1} = 0.059\ eV$ and

${E_R}^{v} = 0.015\, eV$, the first conduction band and valence band respectively. In Supplementary Figure 8 we demonstrate the TPA spectrum for increasing values of the Rashba coefficient α_c subject to the constraint $\alpha_c = 2\alpha_v$ for the pump polarization ε perpendicular and parallel to the direction of inversion symmetry breaking, z. We observe that for both directions of pump polarization, the overall magnitude of β increases for increasing value of the Rashba parameter. This is a result of the increased number of intermediate states that contribute to the summations of the form of equation S8. Additionally, we can see that a new band emerges for large values of α_c that peaks at lower energy compared to the spectra corresponding to bands without Rashba splitting. Lastly, we observe much stronger TPA at the band edge due to the disruption of the parity selection rule for states near the high-symmetry points in the BZ, consistent with that observed in Figure 4a of the main text.

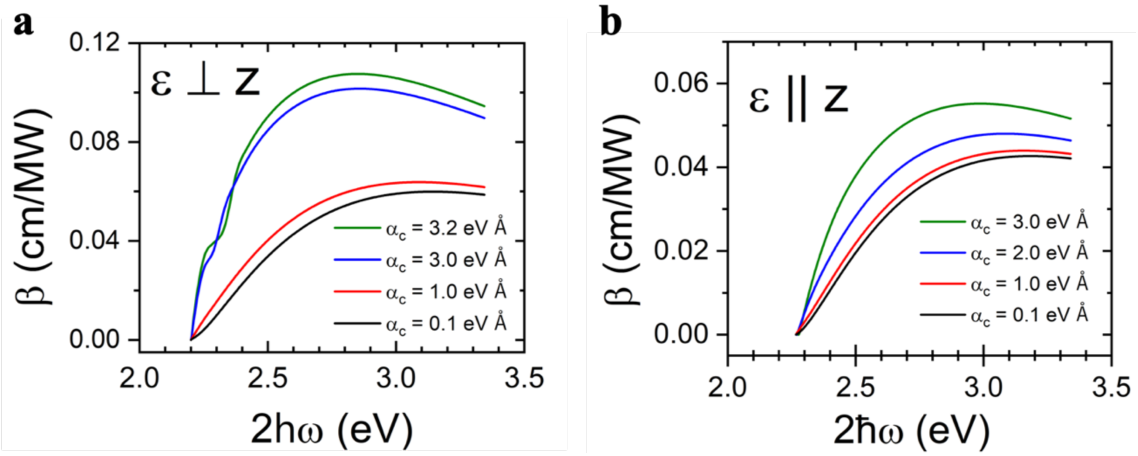

Supplementary Figure 8. **| TPA spectrum of $MAPbBr_3$ including the Rashba effect with final states limited to the first conduction band.** TPA coefficient vs. photon energy for increasing values of the Rashba coefficient α_c subject to the constraint $\alpha_c = 2\alpha_v$ for the pump polarization ε **a** perpendicular to and **b** parallel to the direction of inversion symmetry breaking, z.

On the other hand, when there is a significant discrepancy between the VB and CB effective masses, and consequently, in the Rashba energies between the VB and CB, the transitions $W_{(3,1)}$ and $W_{(3,2)}$ become prominent because the energy onsets for these transitions occur at finite **k**, the result is a sharp onset as $2\hbar\omega$ becomes large enough to connect bands 2 and 3 first and then at higher energy bands 1 and 3. This accounts for the occurrence of the sharp peaks found in $MAPbI_3$ as shown in Figure 4d. The effective masses are to $m_e = 0.46m$ and $m_h = 0.10m$. The

fitting results in values for the Rashba splitting energy of $E_R{}^{c1} = 0.332\ eV$ and $E_R{}^{v} = 0.015\ eV$, the first conduction band and valence band respectively. For the transitions with final states residing in the second conduction band, we have used m_e = 0.40m and obtained $E_R{}^{c2} = 0.240\ eV$. We note a wide range of parameters may satisfy the requirements and thus we have constrained the calculations to the condition $\alpha_R{}^{c} \approx 2\alpha_R{}^{v}$. The conditions for TPA arising from the two conditions described above are summarized in Supplementary Figure 9.

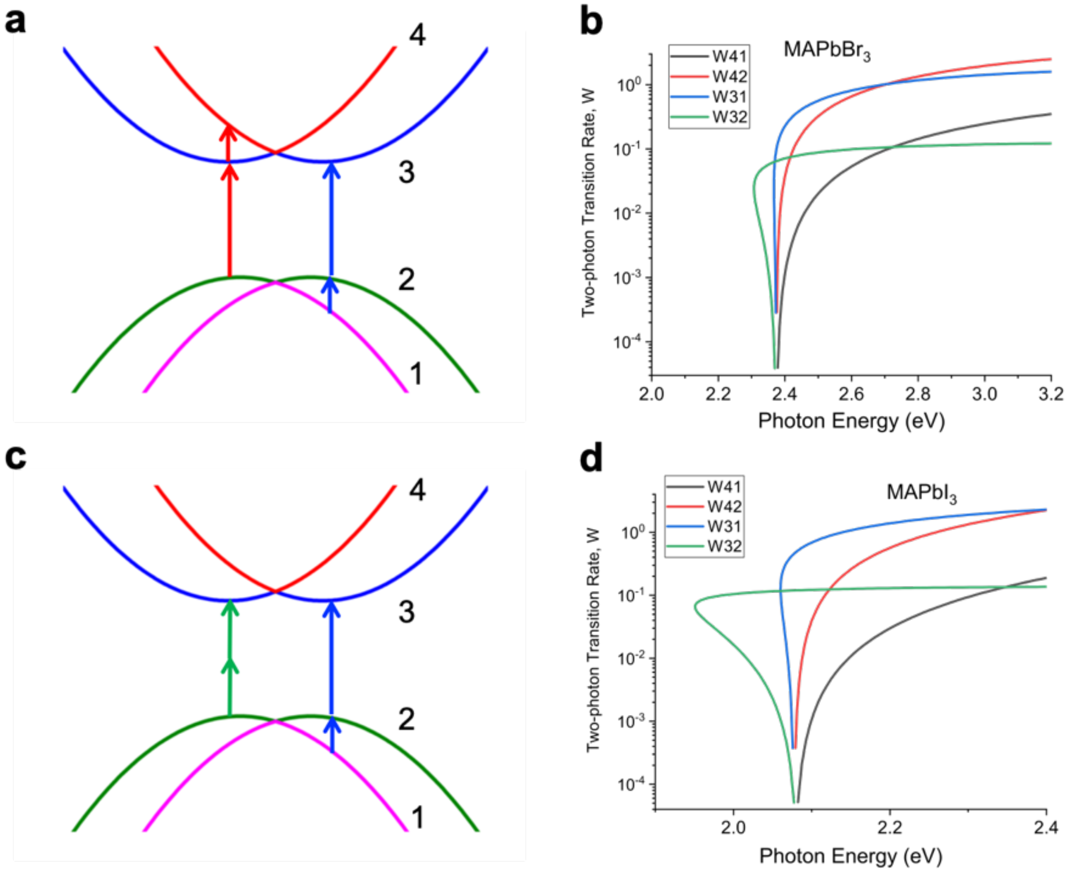

Supplementary Figure 9. | Direct calculation of the two-photon transition rates and resulting dominant contributions. **a** The dominant transitions that contribute to TPA when Rashba energies are comparable represented as the double arrows, between the Rashba-split bands labeled in order of increasing energy. **b** The two-photon transition rates, W, calculated as a function of the photon energy (determined by the energetic difference between respective bands for the parameters used to derive the model fitting for MAPbBr3 namely, $m_e = m_h$ = 0.1m, $E_R{}^{c} = 0.060\ eV$ and $E_R{}^{v} = 0.015\ eV$. **c** and **d** Same as in **a** and **b** but for the parameters used to derive the model fitting for MAPbI3 namely, m_e = 0.46m and m_h = 0.1m, $E_R{}^{c} = 0.332\ eV$ and $E_R{}^{v} = 0.015\ eV$.

Supplementary Note 4. Relation Between Two-Photon Photoluminescence of Perovskite Crystals and Absorption Onset of Perovskite Thin Films

Because absorption measurements of single crystals are difficult and unreliable, we have chosen to compare the TP-PLE to the absorption spectrum of a thin film of the same material. As was noted however, the band-gap at the surface of the crystal is apparently higher than that of the bulk. In Supplementary Figure 10 we compare the one and two-photon excited PL to the same thin film absorption spectra shown in Figure 3 of the main text. The one-photon excited PL band agrees very well with the thin film absorption band-edge feature, indicating the surface band-gap of crystals is similar to the band gap of thin films. Therefore, it is possible that we overestimate the band-gap in our comparison, but our conclusions, in particular, the absence of two-photon exciton resonances at the band-edge, are not affected by this overestimation.

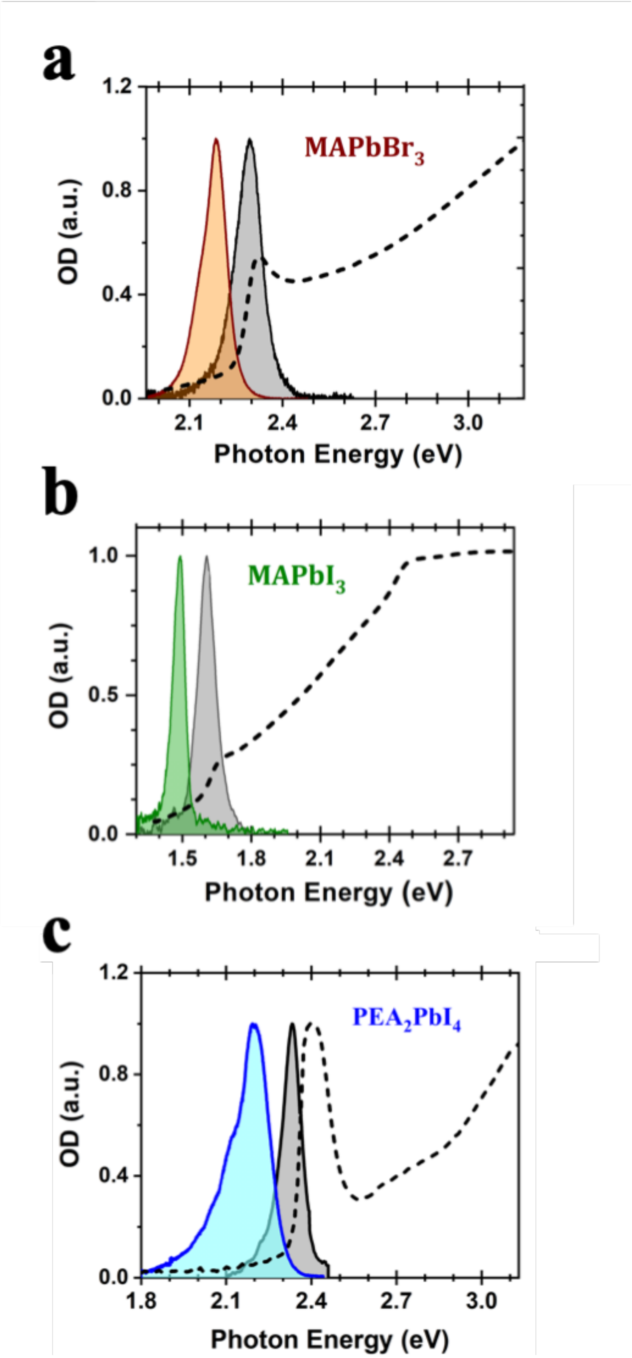

Supplementary Figure 10. | Comparison of Two-photon photoluminescence, one-photon photoluminescence of perovskite crystals and absorption of perovskite thin films. a PL spectra of $MAPbBr_3$ recorded with one-photon (grey) and two-photon excitation (orange) compared to absorption of a thin film (dashed line). **b** PL spectra of $MAPbI_3$ recorded with one-photon (grey) and two-photon excitation (green) compared to absorption of a thin film (dashed line). **c** PL spectra of PEA_2PbI_4 recorded with one-photon (grey) and two-photon excitation (blue) compared to absorption of a thin film (dashed line).

Supplementary Note 5. One-photon photoluminescence excitation spectra of perovskite crystals

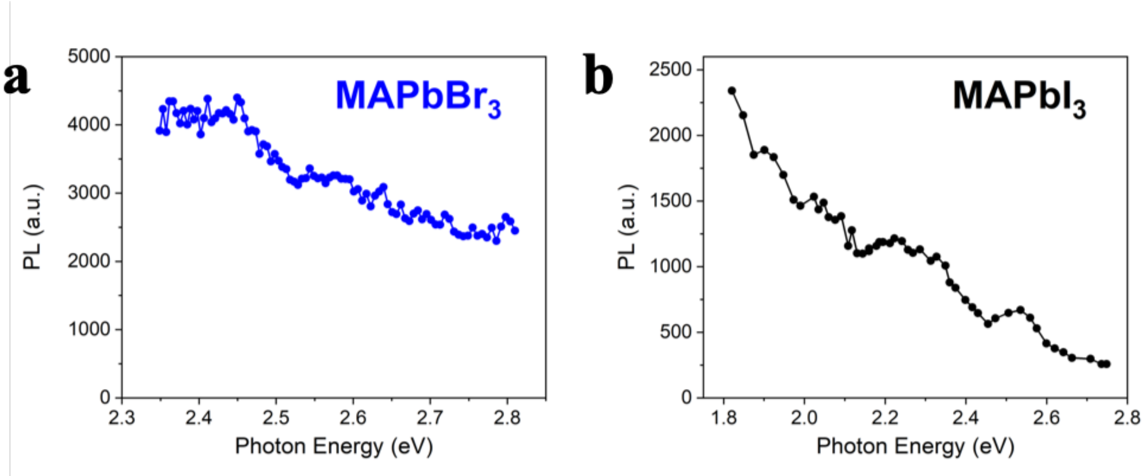

Supplementary Figure 11. | One-photon photoluminescence excitation spectra of perovskite crystals. One-photon photoluminescence spectra of **a** $MAPbBr_3$ and **b** $MAPbI_3$ crystals.

In Supplementary Figure 11, we show the photoluminescence excitation spectra, PLE obtained for photon energies above the optical gap, E_G. Compared to the TP-PLE shown in the main text, these spectra are relatively featureless, exhibiting mainly a trend of decreasing PL with increasing photon energy attributed to the decreased penetration depth of higher energy photons and a corresponding increased contribution of non-radiative surface recombination.

Supplementary Note 6. Calculation of Two-Photon Absorption Spectra Including Excitonic Effect

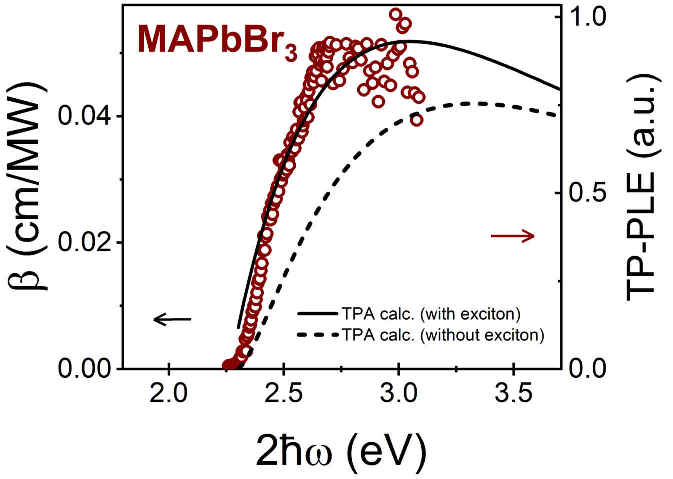

Supplementary Figure 12. | Effect of excitonic enhancement of Two-Photon Absorption. Comparison between the TP-PLE spectrum and theoretical calculations for the TPA response of $MAPbBr_3$ with exciton (solid line) and without exciton effects (dashed line).

The direct resonances of the lowest lying exciton states were discussed in Supplementary Note 1. In addition to this affect, the Coulomb interaction also modifies the wavefunction of the band states. Exciton effects were included using a Green's function approach assuming a hydrogenic spectrum

$$E_n = E_g - \left(\frac{\mu e^4}{2\epsilon_\infty^2 \hbar^2}\right)\frac{1}{n^2} \tag{S11}$$

Where n is the principle quantum number, ϵ_∞ is the high frequency dielectric constant and μ is the reduced mass of the exciton, $1/\mu = 1/m_e + 1/m_h$. The exciton binding energy is 30 meV.

Supplementary Figure 12 shows the calculated TPA spectrum for the final states limited to the first conduction band both with and without the exciton effect included. With excitonic effects included, $\beta(\omega)$ response shows an increase in oscillator strength starting at $2\hbar\omega = E_g$, and a red-shift of the maximum in β (to $2\hbar\omega \approx 1.2E_g$) compared to the spectrum without excitonic. The need to include excitonic enhancement to model the TPA band feature was also observed in $MAPbCl_3$[4].

Supplentary Note 7. Electroabsorption of Perovskite Thin Films

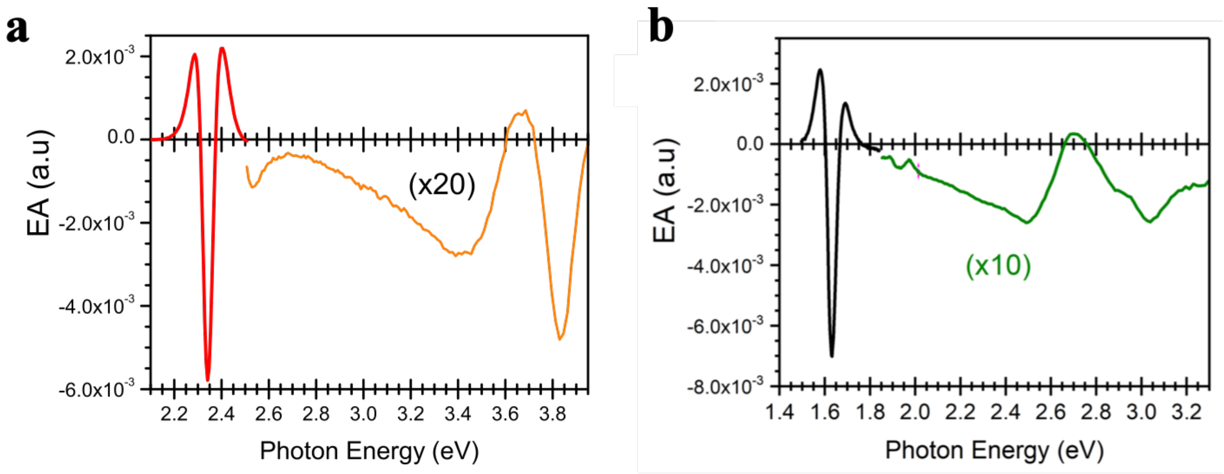

Supplementary Figure 13. | Electroabsorption of perovskite thin films. **a** The electroabsorption spectrum of thin film $MAPbBr_3$ at an applied field of 325 kV/cm. The high-energy part of the spectrum (orange line) has been multiplied by a factor of 20. **b** Same as in (**a**) for thin film $MAPbI_3$. The high-energy part of the spectrum (green line) in this case has been multiplied by a factor of 10.

Electroabsorption measurements were performed using interpenetrating electrodes of 30µm width separated by 20µm gaps formed by deposition of Ti/Au on sapphire followed by a standard lithography procedure and used as substrates. Thin films of perovskites were then deposited on these substrates using the procedures described above. Electric field was applied across the electrodes using a 1.017 kHz square wave voltage signal from a function generator amplified by a step-up transformer. Light from either a Xenon or Tungsten lamp were first dispersed by 1/4m spectrometer and focused on the sample, with the transmission subsequently detected by a Si photodiode and lock-in amplifier referenced to the AC voltage frequency. The sample was housed in a He-exchange cold-finger cryostat with optical windows and electrical terminals during the measurement and held at a pressure of 50mTorr. All measurements were performed at room temperature.

In Supplementary Figure 13, the oscillatory features demonstrate the derivative of absorption due to the exciton and band states, respectively. The lower energy features in Supplementary Figures

13 a and b thus demarcate the energetic location of the exciton states and first VB → CB1 transition. The higher energy oscillation in Supplementary Figure 13a occurs at 3.4 eV indicating the VB → CB2 transition for $MAPbBr_3$. This occurs outside the energetic range of the TP-PLE measurements provided in the main text and thus preclude the inclusion of additional bands in modeling the spectrum. On the other hand, the higher energy oscillation in Supplementary Figure 13b occurs at 2.5 eV indicating the VB → CB2 transition for $MAPI_3$, which is within the experimental TP-PLE range, thus necessitating the inclusion of the second band in modeling the spectra of this compound.

Supplementary Note 8. Comparison of Room Temperature and Low Temperature Two-Photon Photoluminescence

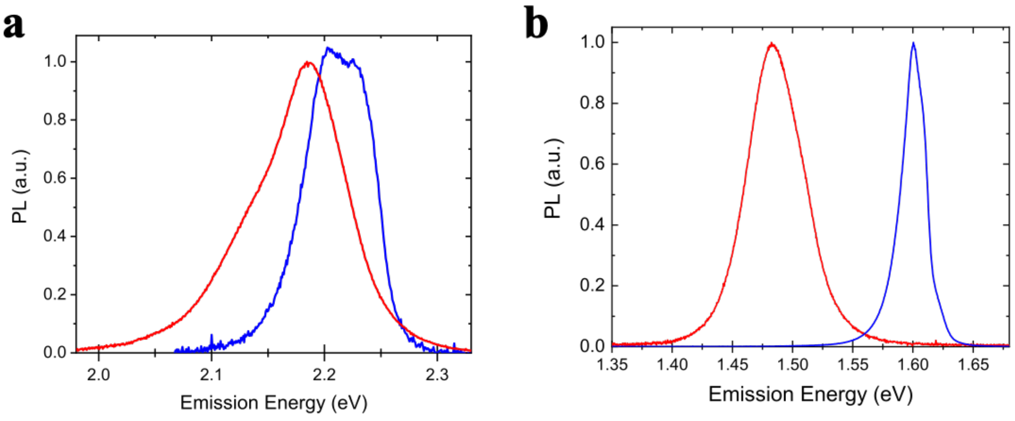

Supplementary Figure 14. | Comparison of Room Temperature and Low Temperature Two-Photon Photoluminescence. **a** Two-photon photoluminescence of $MAPbBr_3$ crystal at room temperature (red) and 50 K (blue). **b** Two-photon photoluminescence of $MAPbI_3$ crystal at room temperature (red) and 77 K (blue).

Supplementary Figure 14a shows TP-PL spectrum from the same crystal of $MAPbBr_3$ at room temperature (RT) and 50K, respectively. The TP-PL at 50K peaks at higher energy at 50 K showing the optical band gap is higher in energy at this temperature in the orthorhombic phase than at RT in the cubic phase. Thus, the lower energy onset of TP-PLE in Figure 4a in the main text shows that the two-photon absorption spectrum of $MAPbBr_3$ is more strongly allowed in the vicinity of the optical gap (at $2\hbar\omega \approx E_G$) due to the effect of broken inversion symmetry. Similarly, Supplementary Figure 14b compares the TP-PL spectrum from the same $MAPbI_3$ crystal at RT and liquid nitrogen temperature. The TP-PL spectrum at low temperature blueshifts by 0.12 eV. Thus, the comparable onsets of the TP-PLE spectrum of $MAPbI_3$ at the two temperatures indicates a redshifted onset of the TPA spectrum at low temperature.

<u>**Supplementary Note 9. Correction for spectrally nonuniform TP-PL response**</u>

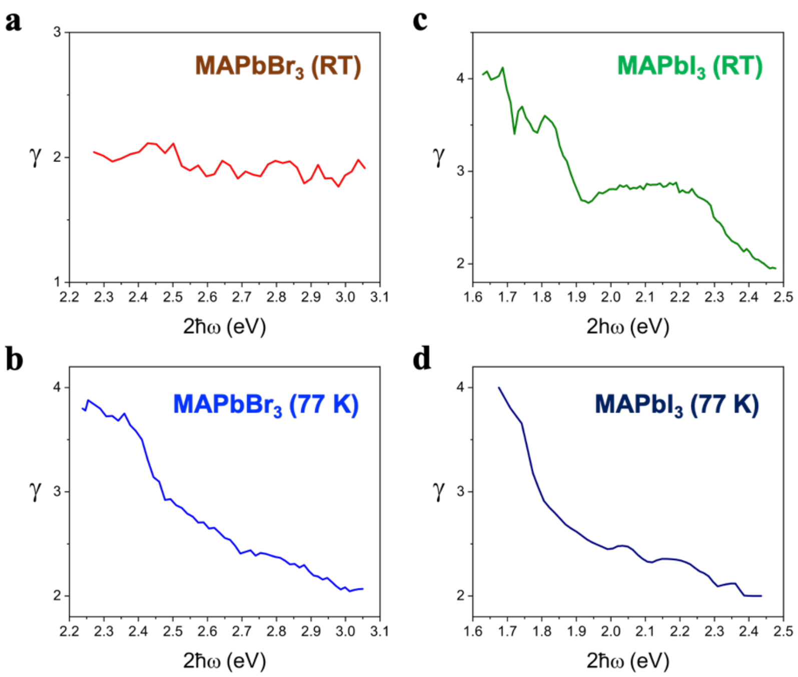

Supplementary Figure 15. | Intensity dependence of TP-PL of perovskite crystals across the excitation spectral range. a-d The experimentally determined pump dependence exponent, γ, as defined by the relation $PL \propto {I_p}^{\gamma}$ where I_p is the pump intensity is shown vs. twice the two-photon excitation energy $2\hbar\omega$ for the perovskite crystals $MAPbBr_3$ at both room temperature (**a**) and low-temperature (**b**) and $MAPbI_3$ at both room temperature (**c**) and low-temperature (**d**).

Only for $MAPbBr_3$ at room temperature we do observe the quadratic pump dependence expected for two-photon absorption and linear conversion of absorbed photons to emitted photons. For $MAPbI_3$ at both temperatures as well as $MAPbBr_3$ at low temperature we observe that the TP-PL depends on the pump pulse energy in a super-quadratic manner until $2\hbar\omega$ is much higher than the band-gap. These results suggest that the conversion of absorbed photons to emitted photons is quadratic near the bad-gap. The super-linear photoluminescence intensity dependence has been observed in one-photon photoluminescence measurements of HOIP[5] and can be attributed to exciton recombination that occurs in the presence of donor or acceptor impurity states in the gap, and is thus evidence of ionic defects and impurity states[6].

Photoluminescence Excitation (PLE) is only equivalent to the absorption spectrum when the PL is a linear function of the absorbed photon density. Then the PLE is described by,

$$PLE(\omega) = A(\omega)\frac{I(\omega)}{\hbar\omega} \quad \text{(S12)}$$

where $A(\omega)$ is the absorption spectrum and $I(\omega)$ is the incident intensity. Then the PLE normalized by the incident intensity gives the absorption. If the PLE response is not linear but depends on the absorbed photon density to some power, r, then we have

$$PLE(\omega) = \left[A(\omega)\frac{I(\omega)}{\hbar\omega}\right]^r \tag{S13}$$

In this case, we can find $A(\omega)$ by measuring the PLE spectrum at different intensities and dividing one by the other. For example, suppose we measure PLE with intensities of $I_1(\omega)$ and $I_2(\omega)$. Then we have

$$\frac{PLE_1(\omega)}{PLE_2(\omega)} = \frac{[A(\omega)I_1(\omega)]^r}{[A(\omega)I_2(\omega)]^r} = \left[\frac{I_1(\omega)}{I_2(\omega)}\right]^r \tag{S14}$$

This allows for the determination of r or $r(\omega)$ by

$$\log\left[\frac{PLE_1(\omega)}{PLE_2(\omega)}\right] = r(\omega)\log\left[\frac{I_1(\omega)}{I_2(\omega)}\right] \tag{S15}$$

We can then obtain the absorption spectrum via correction:

$$A(\omega) = \frac{\hbar\omega[PLE(\omega)]^{1/r(\omega)}}{I(\omega)} \tag{S16}$$

For two-photon absorption, in the ideal case the PLE depends on the square of the intensity: $PLE(\omega) = \beta(\omega)(I^2(\omega)/2\hbar\omega)$, where $\beta(\omega)$ is the TPA spectrum. However, in the non-ideal case we have

$$PLE(\omega) = [\beta(\omega)I^2(\omega)]^r \tag{S17}$$

By the same method as for the one photon PLE above we may obtain

$$\log\left[\frac{PLE_1(\omega)}{PLE_2(\omega)}\right] = r(\omega)\log\left[\left(\frac{I_1}{I_2}\right)^2\right] \tag{S18}$$

and

$$\beta(\omega) = \frac{[PLE(\omega)]^{1/r}}{I^2(\omega)} \tag{S19}$$

We have accordingly used the experimentally determined pump dependent exponent ($\gamma = 2r$) shown in Supplementary Figure 15 to correct the measured TP-PLE spectra and extract the TPA spectrum $\beta(\omega)$ for the case of $MAPbBr_3$ at low -temperature and for $MAPbI_3$ at both room-temperature and low-temperature.

Supplementary Note 10. PL and TP-PLE of Mixed-halide crystals

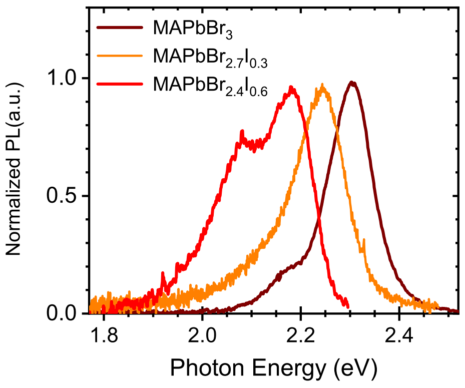

Supplementary Figure 16. | One-photon photoluminescence spectra of mixed-halide crystals and $MAPbBr_3$ as denoted.

Supplementary Figure 16 shows the one-photon photoluminescence spectra of the mixed halide crystals used in this study compared to that of $MAPbBr_3$. The peaks of the PL spectra are used to determine the position of the exciton at 2.24 eV for $MAPbBr_{2.7}I_{0.3}$ and 2.18 eV for $MAPbBr_{2.4}I_{0.6}$.

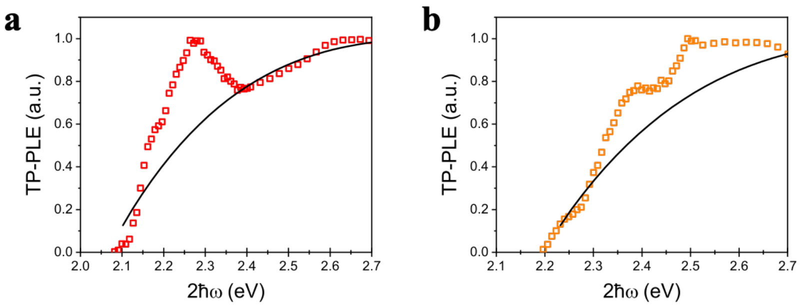

Supplementary Figure 17. | Comparison of TP-PLE of mixed halide perovskites to the TPA model including exciton enhancement. Comparison of the TP-PLE spectrum of **a,** $MAPbBr_{2.7}I_{0.3}$ and **b,** $MAPbBr_{2.4}I_{0.6}$ crystals to the one-band TPA model with exciton enhancement.

Supplementary Figure 17 shows the TP-PLE spectra from Fig. 5a of the main text compared to the one-band model for two-photon absorption with exciton enhancement described in Supplementary Note 6. Unlike the case for $MAPbBr_3$, the model cannot account for the additional band at low-energy observed in the mixed-halide perovskite crystals.

S11. XRD of Perovskite Crystals

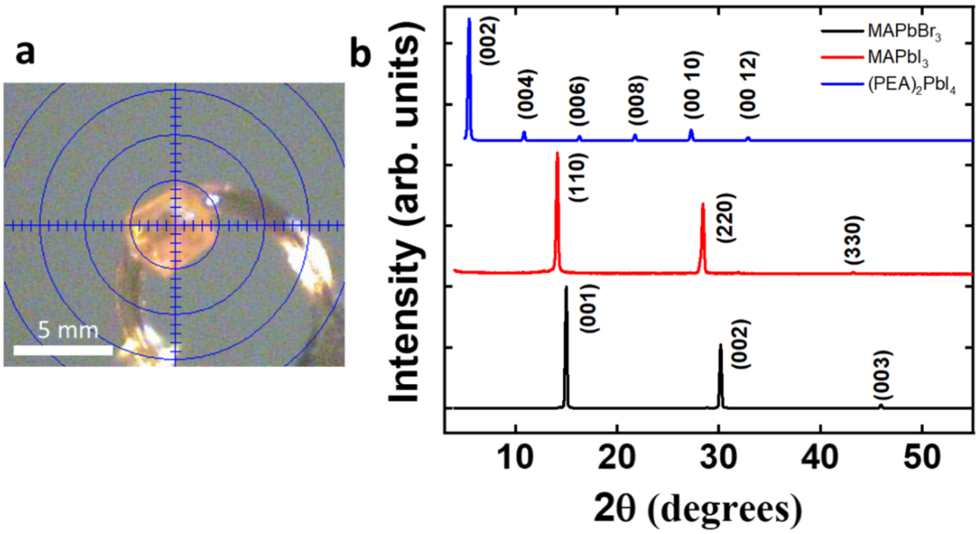

Supplementary Figure 18. **| XRD of Perovskite Crystals. a** Optical microscopy image showing the size of the single crystal used for the XRD experiments. The crosshair indicates the position of the X-ray beam. **b** XRD patterns for $MAPbBr_3$, $MAPbI_3$, $(PEA)_2PbI_4$ single crystals.

Supplementary Figure S18 shows indexed X-ray diffraction (XRD) patterns for $MAPbBr_3$ and $MAPbI_3$ single crystals. For a $MAPbBr_3$ single crystal, the diffraction peak assignments corroborate the formation of a cubic structure with its most intense diffraction peaks corresponding to reflections associated with the (001), (002), and (003) crystallographic planes at $2\theta = 14.9^\circ$, 30.2°, and 43.2°, respectively. In the case of a $MAPbI_3$ single crystal, we observe the formation of a tetragonal structure with its most intense diffraction peaks corresponding to reflections associated with the (110), (220), and (330) crystallographic planes at $2\theta = 14.1^\circ$, 28.2°, and 43.2°, respectively. The XRD patterns presented for both single crystals also demonstrate their anisotropic nature and high quality since only intensities associated to the $\{00l\}$ and $\{hk0\}$ family of planes for $MAPbBr_3$ and $MAPbI_3$ are observed, respectively. For $(PEA)_2PbI_4$, we observe the formation of highly oriented $(00l)$ peaks associated with the (002), (004), (006), (008), (00 10), and (00 12) at $2\theta = 5.4^\circ$, 10.9°, 16.3°, 21.8°, 27.3°, and 32.9°, respectively.

Supplementary References

1. Koster, G. F, Dimmock, J. O, Wheeler, R. G. & Statz, H. *Properties of the Thirty-Two Point Groups*. (MIT Press, 1963).
2. Boyd, R. W. *Nonlinear Optics* (Academic Press, 1992).
3. Yu, Z.-G., Effective-mass model and magneto-optical properties in hybrid perovskites *Sci. Rep.* **6**, 28576 (2016).
4. Ohara, K. et al. Excitonic enhancement of optical nonlinearities in perovskite $CH_3NH_3PbCl_3$ single crystals. *Phys. Rev. Mater.* **3**, 111601(R) (2019).
5. Phuong, L. Q. et al. Free Carriers versus Excitons in $CH_3NH_3PbI_3$ Perovskite Thin Films at Low Temperatures: Charge Transfer from the Orthorhombic Phase to the Tetragonal Phase. *J. Phys. Chem. Lett.* **7**, 2316 – 2321 (2016).
6. Schmidt, T., Lischka, K., & Zulehner, W. Excitation-power dependence of the near-band-edge photoluminescence of semiconductors. *Phys. Rev. B.* **45**, 8989 – 8994 (1992).
